# Mixing properties of coronary infusion catheters assessed by *in vitro* experiments and computational fluid dynamics

**DOI:** 10.1093/ehjdh/ztae033

**Published:** 2024-05-16

**Authors:** Annemiek de Vos, Sophie Troost, Anke Waterschoot, Nico Pijls, Marcel van ‘t Veer

**Affiliations:** Department of Cardiology, Catharina Hospital, Eindhoven, The Netherlands; Department of Biomedical Engineering, Eindhoven University of Technology, Eindhoven, The Netherlands; LifeTec Group, Eindhoven, The Netherlands; Department of Cardiology, Catharina Hospital, Eindhoven, The Netherlands; Department of Cardiology, Catharina Hospital, Eindhoven, The Netherlands; Department of Biomedical Engineering, Eindhoven University of Technology, Eindhoven, The Netherlands

**Keywords:** Coronary physiology, Microvascular resistance, Continuous thermodilution, Computational fluid dynamics

## Abstract

**Aims:**

Continuous infusion thermodilution is an established technique for the assessment of absolute coronary blood flow and microvascular resistance due to its proven accuracy and reproducibility. However, for this technique to yield reliable measurements, direct and homogenous mixing of injected saline and blood is mandatory. This study aimed to assess and compare the mixing properties of two different microcatheters, namely the Rayflow® (with sideholes for infusion) and the Finecross® catheter (single end-hole for infusion), which are commonly used in the catheterization laboratory.

**Methods and results:**

The study employed three different methods to evaluate the mixing properties of the catheters. Firstly, a qualitative assessment of mixing was performed using ink injections in an *in vitro* bench model of a coronary artery. Secondly, in analogy to the human catheterization laboratory, mixing properties over the length of the coronary artery were assessed semi-quantitatively by temperature measurements in the bench model. Lastly, a quantitative assessment was performed by 3D computational fluid dynamics, where the standard deviation and entropy ratio of the temperature over the cross-section in the coronary artery model were calculated for both catheters.

**Conclusion:**

All three evaluation methods demonstrated that the Rayflow catheter’s specific design leads to a more optimal, homogeneous mixture of blood and saline over both the cross-section and length of a coronary vessel, as compared with the standard end-hole catheter.

## Graphical Abstract

**Graphical Abstract:**
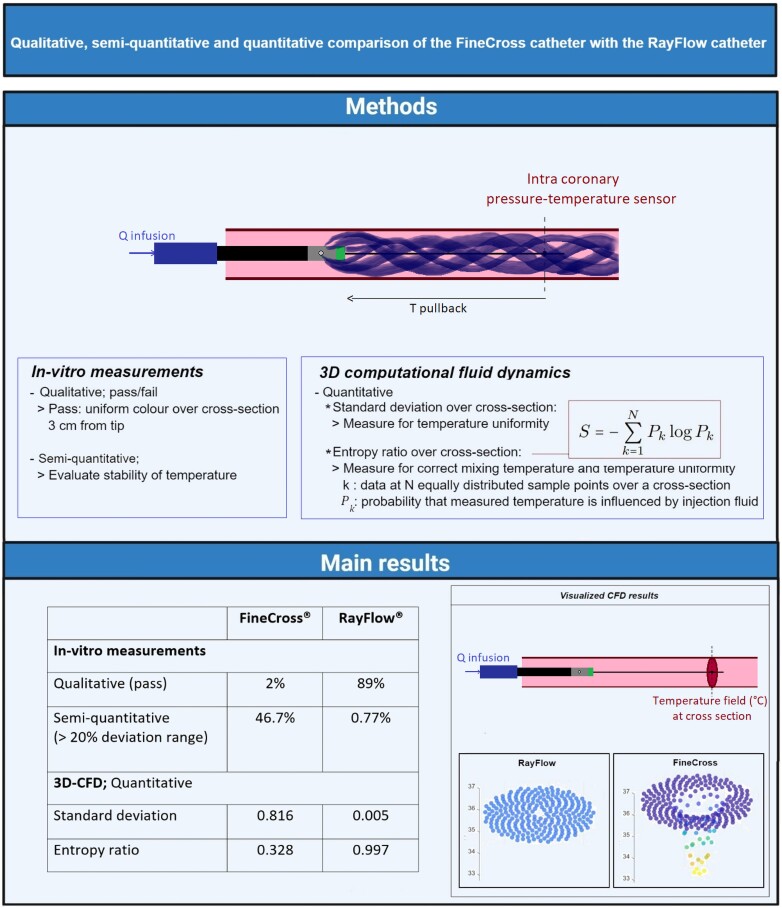
Methods and main results are shown for *in vitro* measurements and 3D computational fluid dynamics.

## Introduction

Over the past decade, continuous infusion thermodilution has been increasingly used to assess absolute coronary blood flow and microvascular resistance^1–3^ and is currently applied in ∼80 heart centres in Europe.

An infusion catheter is required for the application of this technique to ensure complete mixing of blood and infused saline. Direct and homogeneous mixing along the length of the coronary artery is a prerequisite for obtaining reliable measurements. A catheter that is used for these types of measurements is the Rayflow catheter (HexaCath, Paris, France).^4^ The distal end of this monorail infusion catheter contains a central lumen for the pressure temperature guide wire surrounded by an infusion lumen containing four outer side holes close to the tip of the catheter and two inner side holes a few millimeters proximal to its tip to measure the temperature of the infused saline.^1,4,5^ Using ink injections, Van ‘t Veer et al.^4^ have shown qualitatively that the mixing properties differ between the Rayflow catheter and end-hole catheters such as the Finecross infusion catheter (Terumo, Tokyo, Japan). However, no systematic or quantitative assessments have been done to compare the mixing properties of the Rayflow catheter, with the pressure temperature wire inside the infusion catheter, and a conventional and widely used end-hole infusion catheter, with the pressure temperature wire alongside the infusion catheter. Moreover, no information has been obtained yet to quantify the homogeneity of the mixing of infused saline and blood over the length of the vessel and over the cross-section of the vessel. Computational fluid dynamic (CFD) simulations can provide detailed spatial and temporal information in a model describing continuous infusion thermodilution for absolute coronary blood flow measurements that cannot be obtained by *in vitro* experiments. The purpose of this study is to compare the commonly used Rayflow catheter and the Finecross end-hole infusion catheter with respect to mixing properties using both *in vitro* experiments and by means of 3D CFD simulations.

## Methods

### *In vitro* setup

**Figure 1:**
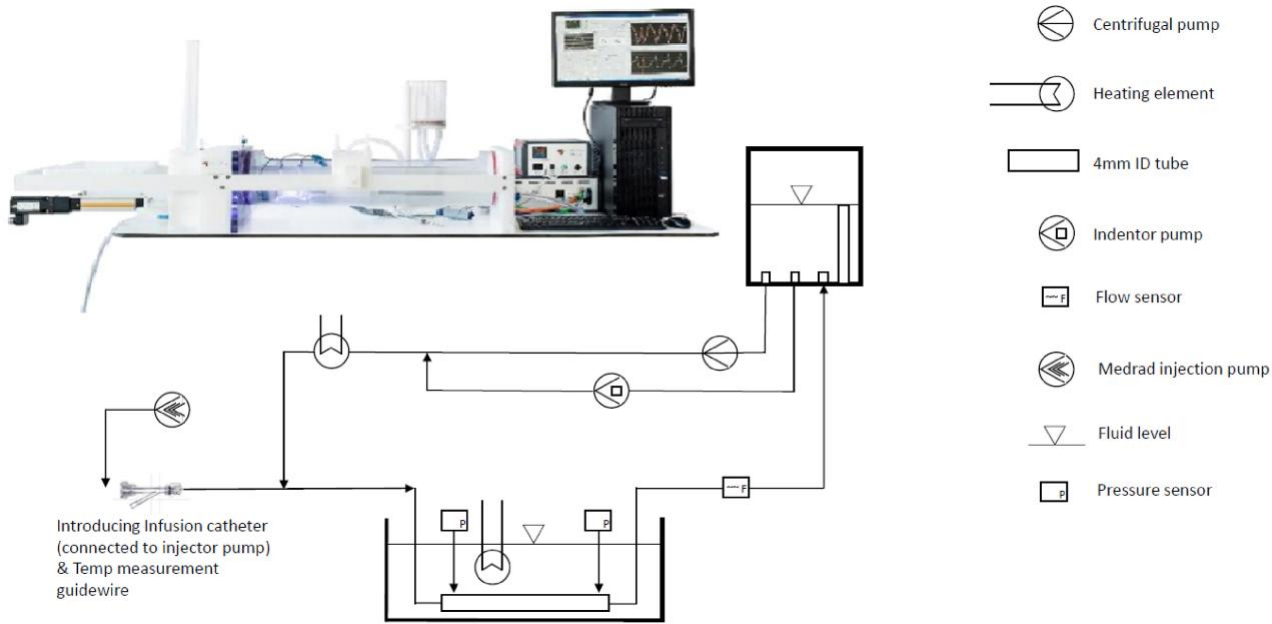
Illustration of the bench model mimicking the coronary artery and physiology and schematic display of the setup (LifeTec Group, Eindhoven, The Netherlands).

A bench model mimicking the coronary circulation was set up by creating a pulsatile flow in a 4 mm diameter tube (*Figure 1*) to compare the Rayflow infusion catheter and the Finecross infusion catheter. A blood-mimicking medium was used that consisted of 0.05% Xanthan gum/demi water and was heated to 37°C having a viscosity of 3.71 mPa, equal to the viscosity of blood with a haematocrit of 34%.^6^ Low (50 mL/min, LOW), medium (150 mL/min, MED), and high (250 mL/min, HIGH) coronary flow rates were set up to mimic the flow range between resting coronary blood flow and maximum hyperaemic coronary blood flow as encountered in human coronary arteries. Saline at room temperature was infused at rates of 8, 10, 15, 20, 25, and 30 mL/min imitating the range used in the human catheterization laboratory.

All combinations of coronary flow rates and infusion rates yield 18 possibilities (3 × 6). However, in clinical practice, certain combinations of infusion rates and (estimated) coronary flow rates are not used. For example, if coronary flow in a small vessel is expected to be in the range of 50 mL/min, an infusion rate of 20 mL/min or higher will not be used since the infusion flow will comprise almost 50% of the coronary flow, thereby influencing the coronary physiology. Likewise, an infusion rate between 8 and 15 mL/min will not be used at expected coronary flow values of 250 mL/min since that will lead to a suboptimal signal-to-noise ratio. Therefore, the focus of this research was on nine clinically used combinations of coronary blood flow rates and infusion rates as depicted in *Table 1*.

**Table 1:** Overview of clinically relevant combinations as used in the bench model, mimicking LOW coronary blood flow (50 mL/min), MED coronary blood flow (150 mL/min), and HIGH coronary blood flow (250 mL/min), with appropriate infusion rates per category of coronary blood flow

Coronary blood flow	Infusion rates (mL/min)		
**LOW**	**8^a^**	**10**	**15^a^**
**MED**	**15^a^**	**20**	**25**
**HIGH**	**20**	**25^a^**	**30**

The combinations of coronary flow rates and infusion rates indicated with an asterisk (^a^) are assessed with computational fluid dynamics (CFD).

The mixing pattern (i.e. the quality of the mixing of both fluids) was assessed *qualitatively* by visualization of injection of a saline–ink mixture into the coronary blood flow and (*semi-)quantitatively* by measuring temperature of the mixture over a length of 6 cm from the injection site to the distal location of the temperature sensor and by performing CFD analyses.

### Qualitative visual assessment of mixing using ink injections

A saline–ink mixture was injected using an infusion pump (contrast power injector; Medrad, Bayer, USA) connected to the infusion catheters with a Y-connector. These ink ‘clouds’ were filmed, and still frames at 10 s after the start of infusion were stored and independently assessed. Proper mixing (‘pass’) was defined if a uniform colour over the diameter was observed, looking at the vessel from one direction, within 1–3 cm from the tip of the infusion catheter. In all other cases, mixing was defined as suboptimal (‘fail’). For each infusion catheter type, five samples were tested for the coronary flow rates and infusion rates depicted in *Table 1* (5 × 9 combinations) yielding a 2 × 45 binary results. The complete set of combinations and their accompanying results can be found in Appendix 1.

### Semi-quantitative assessment of mixing by temperature measurements

In analogy to the human catheterization, laboratory saline at 23°C (room temperature) was injected into heated blood-mimicking medium at 37°C. A pressure/temperature wire (Abbott, Saint Paul, USA) was pulled back manually from 6 cm distal to the tip of the infusion catheter in 15–30 s. All pullbacks were recorded using Coroventis software (Coroventis, Uppsala, Sweden). The pullback commenced 30 s after saline infusion was started. In case of proper mixing, the temperature *T* of the mixture distal to the infusion site would be uniform along the pullback. A rolling mean was applied on the temperature data with a window of 2 s comparable with clinical practice.

To be able to directly compare each pullback tracing, data were normalized to *N* = 1000 data points to compensate for the differences in pullback times. Then, the temperature values were expressed as the relative deviation (in %) from the mean temperature during the pullback according to:


(1) $$\Delta T_{\mathrm{rel},i}=\frac{T_{i}-{\bar{T}}_{\mathrm{pb}}}{{\bar{T}}_{\mathrm{pb}}}\cdot 100\text{\%}\;\mathrm{for}\;i=1\;\mathrm{to}\;N,$$

where *T_i_* is the temperature at any point during the pullback and the mean of the temperature ${\bar{T}}_{\mathrm{pb}}$ during the pullback was calculated as:


(2) $${\bar{T}}_{\;pb}=\frac{1}{N}\overset{N}{\underset{i=1}{\sum }}\;T_{i}$$

Finally, the percentages of data points that deviate more than 15%, and 20% were calculated for the coronary flow rate and infusion rate combinations as depicted in *Table 1*. Typically, a variability between values for repeated measurements of <20% is considered clinically acceptable.^7^ Two samples were tested for each infusion catheter type yielding 36 recordings. The complete set of combinations and their accompanying results can be found in Appendix 1.

### Quantitative assessment of mixing by 3D computational fluid dynamics

**Figure 2:**
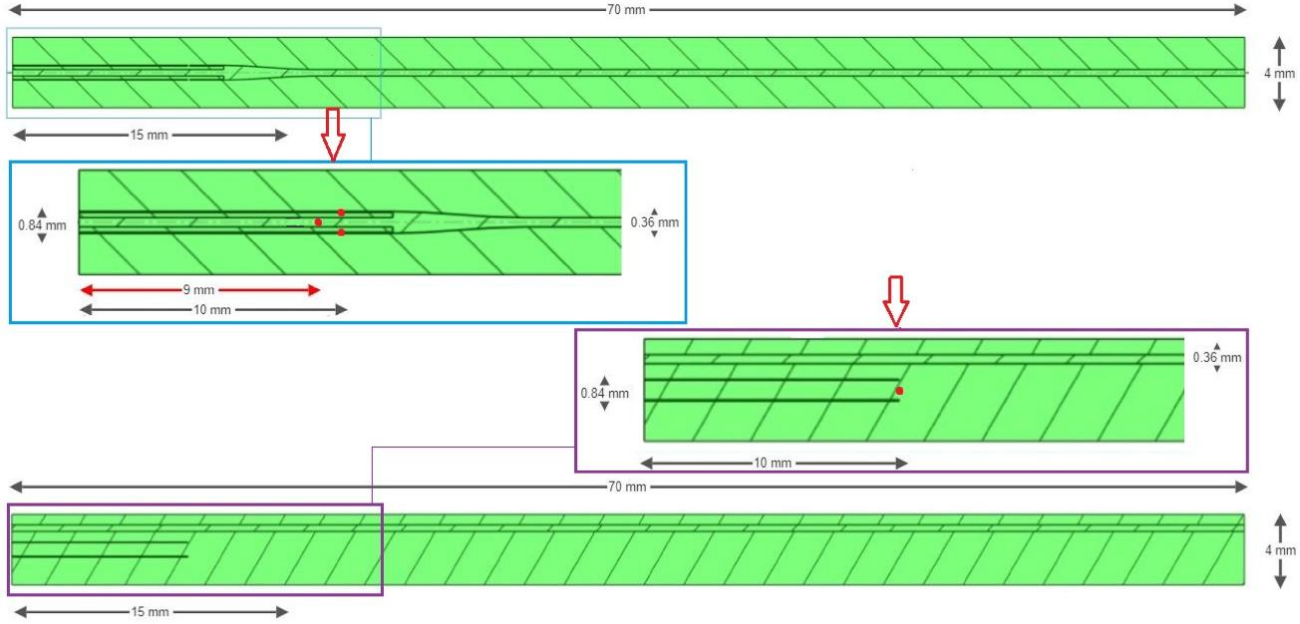
Graphical overview of the CFD geometries for the Rayflow catheter (with the pressure/temperature wire inside the catheter) and the Finecross catheter (with the pressure/temperature wire alongside the catheter) where the upper enlarged frame shows a close-up of the Rayflow geometry and the lower enlarged frame embarks the Finecross geometry. The small dots indicate the positions of the outlets.

3D CFD simulations were performed to quantitatively compare the mixing by the Rayflow catheter and the Finecross catheter in an idealized setting using Ansys Fluent (Canonsburg, Pennsylvania, USA). Meshes were made using Ansys Fluent Meshing (Canonsburg, Pennsylvania, USA). A mesh divides the fluid domain into a large number of elements where the nodes and edges overlap. The element sizes and mesh refinements were imposed by the user (Appendix 2). Both catheters were modelled as straight tubes with an outer diameter of 0.84 mm inside a coronary artery with a diameter of 4 mm and a length of 70 mm. The outlets of the Rayflow (four 100 μm sideholes) and the Finecross (single end hole) were located 10 mm into the artery. The 0.36-mm-thick pressure/temperature wire was modelled in both geometries (inside the Rayflow and alongside the Finecross) as shown in *Figure 2*.

Water was used for both the injected fluid and for the liquid that represents the blood in the simulations. Furthermore, a pulsating coronary blood flow was prescribed at the coronary inlet and the coronary fluid was set to have a temperature of 37°C. Taking into account the heating of the infusion fluid at room temperature while it travels through the catheter inside the body, the temperature was set to 29°C when it leaves the infusion catheters. The inflow of the infusion fluid at the inlet was prescribed to be continuous. Further boundary conditions that were prescribed were the no-slip conditions at all walls and a pressure of 0 mmHg at the coronary outlet.

The continuity, momentum, and energy equations were solved in each node to obtain the pressure, flow, and temperature field throughout the entire domain based on the prescribed inflow and boundary conditions. Two cardiac cycles of 1 s were simulated, and the results were extracted at multiple time steps during the second cardiac cycle. This way, the development of the temperature mixing in the first cycle is negated. *Table 1* shows the combinations of flow rates for which simulations were done indicated by asterisks.

To quantify the mixing, two measures were defined: the standard deviation (SD_t_) and the entropy ratio (*E*) of the temperature. These measures were obtained at multiple cross-sections distal to the infusion site during infusion.

### Standard deviation

**Figure 3:**
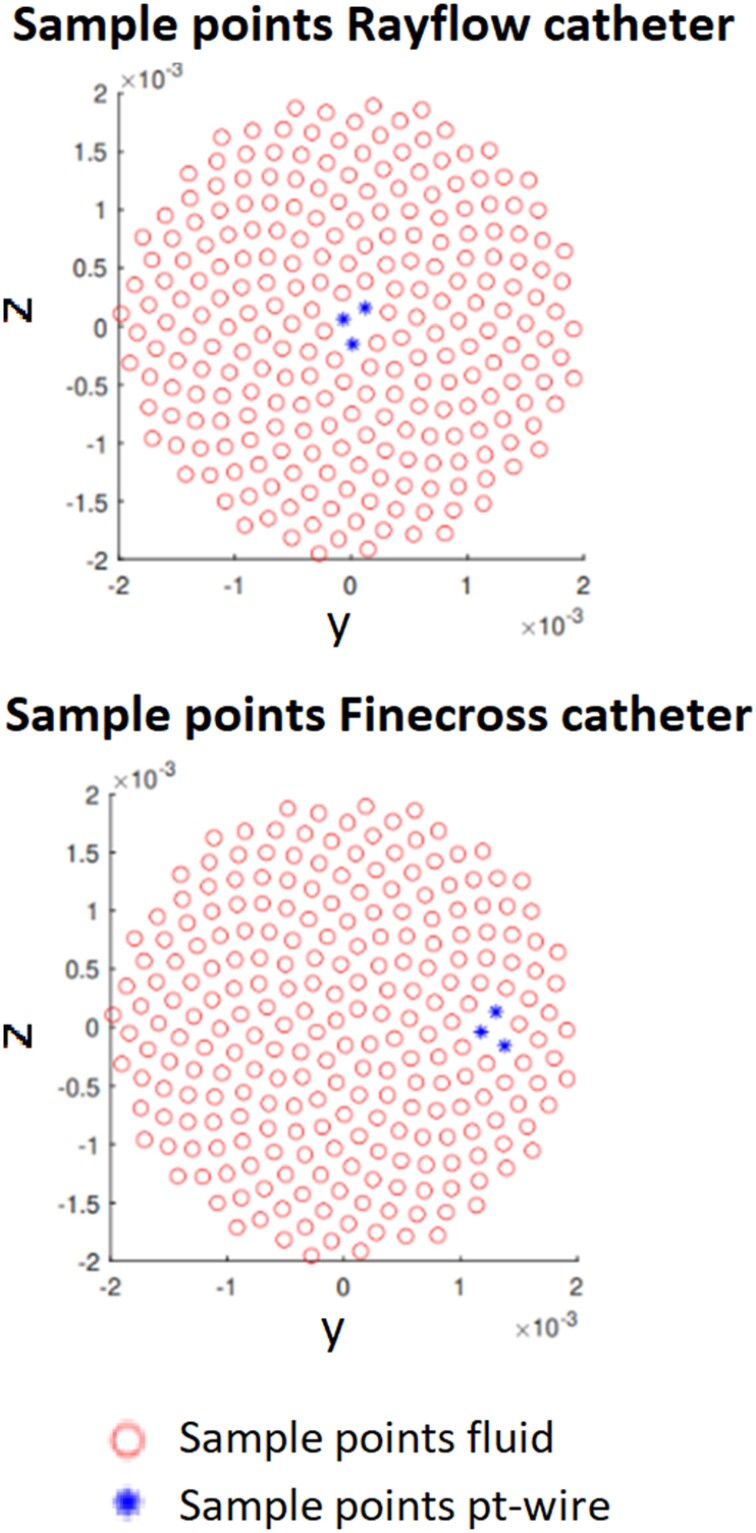
Cross-section sample points for the simulations of the Rayflow catheter (top) and Finecross catheter (bottom). Note that the sample points indicated in asterisks represent the location of the pressure/temperature (pt) wire and are excluded from the time-averaged standard deviation (SD_t_) and the entropy ratio (*E*).

The SD_t_ was calculated based on *n* uniformly distributed locations over a cross-section as depicted in *Figure 3* using:


(3) $$SD_{t}=\sqrt{\frac{\sum_{k}^{n}\;|T_{m,k}-\bar{T}|}{n}},$$

with *T_m,k_* being time-averaged temperature of one cardiac cycle at a specific sample location and $\bar{T}$ the time-averaged mean temperature over the cross-section. An SD_t_ of 0 means that the temperature is uniform over the cross-section.

### Entropy ratio

The entropy ratio measure reflects the homogeneity of the temperature over a cross-section in combination with the expected temperature in case of perfect mixing. The measure is based on the Shannon entropy.^8^ The entropy can be calculated based on the probability that a measured temperature is influenced by the temperature of the injected fluid. This probability is calculated as:


(4) $$P=\frac{T_{b}-T}{T_{b}-T_{i}},$$

with *T*_b_ the temperature of the blood, *T*_i_ the temperature of the infused fluid, and *T* the temperature of the mixture of both following the prescribed coronary blood flow and injection temperature and infusion rate. The normalized entropy over a cross-section can then be calculated by:


(5) $$E=-\frac{\sum_{k=1}^{n}\;P_{k}\log\;P_{k}}{n}$$

with *n* the uniformly distributed locations over a cross-section (*Figure 3*) and *P_k_* the probability at each of these locations. When *E* is equal to 1, the temperature distribution over a cross-section is assumed to be homogeneous.

## Results

### Qualitative visual assessment of mixing using ink injections

**Figure 4:**
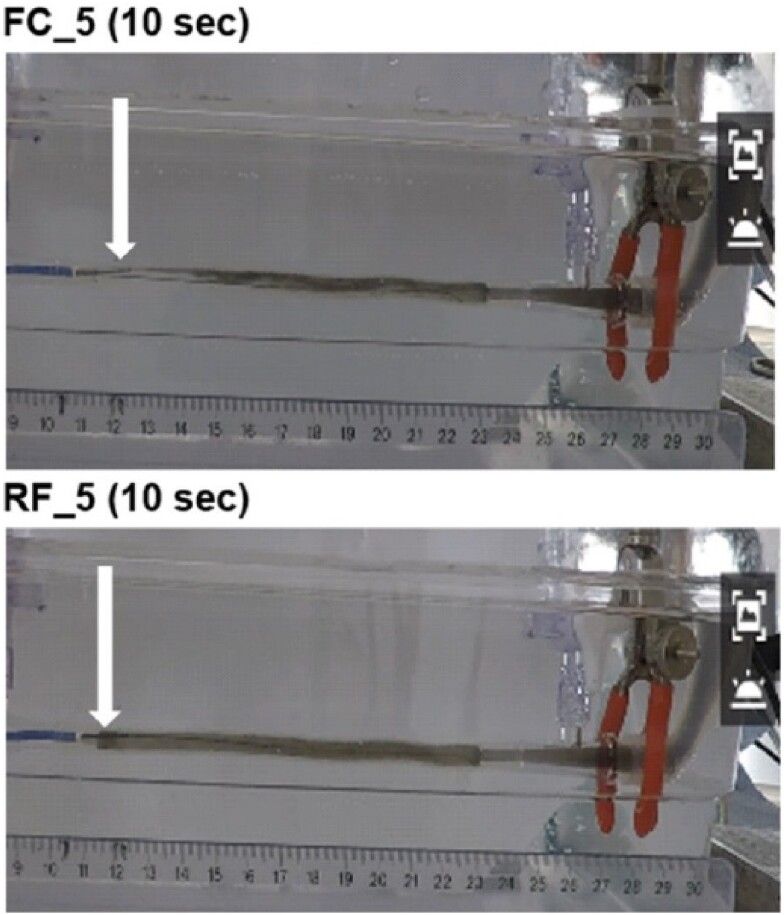
Still frames for both the Finecross (top) and the Rayflow (bottom) at 10 s after the start of the ink injection. The tip of the infusion catheter is indicated by the arrow in each panel, and the distances can be observed from the liner in the bottom. The FC_5_ (top) shows incomplete mixing (‘fail’), whereas the RF_5_ catheter (bottom) shows complete mixing and thus ‘pass’.

Videos were made for five Rayflow and for five Finecross catheters for the coronary flow values and infusion flow values depicted in *Table 1*. All videos were acquired successfully. An example of one still frame for each catheter is shown in *Figure 4*. The pass rate for the Rayflow catheters was 89% (40/45) and showed to be superior to the results of the Finecross where only 2% passed (1/45). The results are stratified for low, medium, and high coronary flow rates in *Table 2*. The results for all combinations are shown in Appendix 1.

**Table 2:** Pass–fail results for clinically relevant combinations of coronary blood flow and infusion rates as depicted in *Table 1*

	LOW		MED		HIGH	
	Fail	Pass	Fail	Pass	Fail	Pass
FC	15	0	14	1	15	0
RF	4	11	1	14	0	15

FC, five tested Finecross catheters; RF, five tested Rayflow catheters.

### Semi-quantitative assessment of mixing by temperature measurements

**Figure 5:**
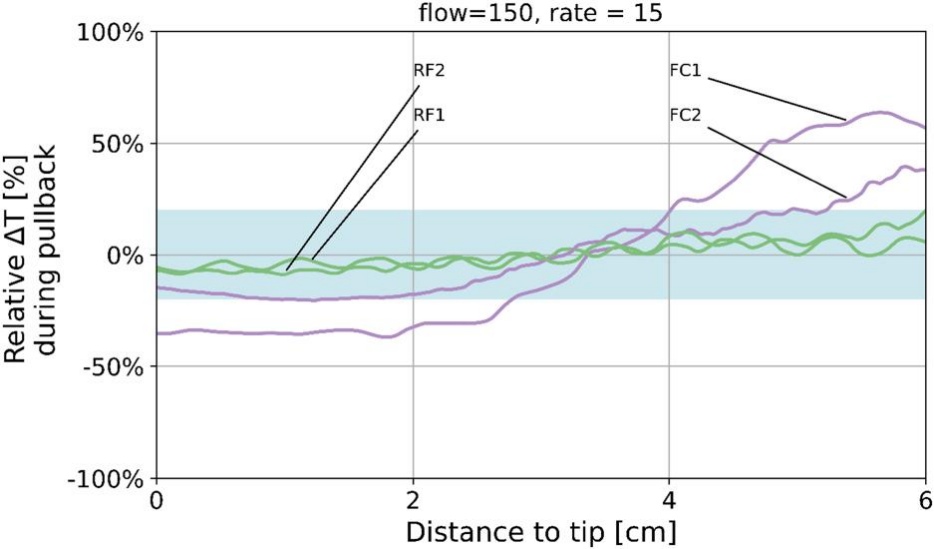
Example of relative deviations of the normalized temperatures Rayflow (indicated by RF1 and RF2) and Finecross (indicated by FC1 and FC2) infusion catheters. On the *x*-axis, the distance to the tip in cm is depicted from 6 cm distal to the tip (6 cm) to the tip itself (0 cm) and on the *y*-axis the relative deviation (in %) with respect to the mean temperature.

All temperature pullback recordings were made successfully for two Rayflow catheters (RF_1_ and RF_2_) and two Finecross catheters (FC_1_ and FC_2_). An example of the normalized relative temperature deviation pullbacks is shown in *Figure 5*. In this case, the relative temperature measured 6 cm distal to the tip is higher compared with the temperature near the tip for the FC catheter, whereas the relative temperatures are rather constant over the length for the RF catheters. The percentage of data points that fall outside 15% and 20% deviation range was 1.1% and 0.77% for the Rayflow catheters and 54.2% and 46.7% for the Finecross catheters.

### Quantitative assessment by 3D computational fluid dynamics

#### Standard deviation of temperature over cross-section

The left part of *Table 3* shows that the time-averaged standard deviation (SD_t_) at the cross-sections at 1.2 cm (*prox*) and 4.4 cm (*dist*) distal to the infusion site for the Rayflow catheter are all smaller than 0.03 and relatively close to 0. This indicates the nearly homogeneous temperature over the cross-section beyond 1.2 cm distal to the infusion site in case of the Rayflow for all coronary flow and infusion rate combinations. The SD_t_ values for the Finecross catheter simulations are two orders of magnitude larger than the SD_t_ values for the Rayflow simulations.

**Table 3:** Results for the time-averaged standard deviation (SD_t_) (left) and the entropy ratio (right) (*E*) for the 3D computational fluid dynamic analyses of the Rayflow (RF) and Finecross (FC) catheters

		RF		FC		RF		FC	
		SD_t_				E			
		Prox	Dist	Prox	Dist	Prox	Dist	Prox	Dist
	**Inf**								
LOW	**8**	<0.01	<0.01	1.07	0.90	0.997	0.980	NA	0.506
LOW	**15**	0.01	0.01	1.13	1.09	0.993	0.984	NA	0.490
MED	**15**	0.01	0.01	1.01	0.82	1.017	0.997	NA	0.328
HIGH	**25**	0.03	0.01	1.03	0.86	1.024	1.006	NA	0.301

The results are obtained at two distances: prox (1.2 cm) and dist (4.4 cm) distal to the infusion sites of the respective infusion catheter.

#### Entropy ratio of temperature over cross-section

The results for the time-averaged Entropy ratio (*E*) at 1.2 (prox) and 4.4 cm (dist) distal to the infusion site show values of *E* close to 1 for all simulations with the Rayflow catheter, described on the right side of *Table 3*. Values of *E* for the Finecross catheter were not calculable for all simulations at 1.2 cm distal to the infusion site because no mixing of the blood and infused fluid had occurred resulting in a numerator of 0 in equation (4), which caused a non-calculable *E* in equation (5). For the Finecross catheter, the maximum value of *E* reached 0.51 at 4.4 cm distal from the outlet indicating incomplete mixing.

**Figure 6:**
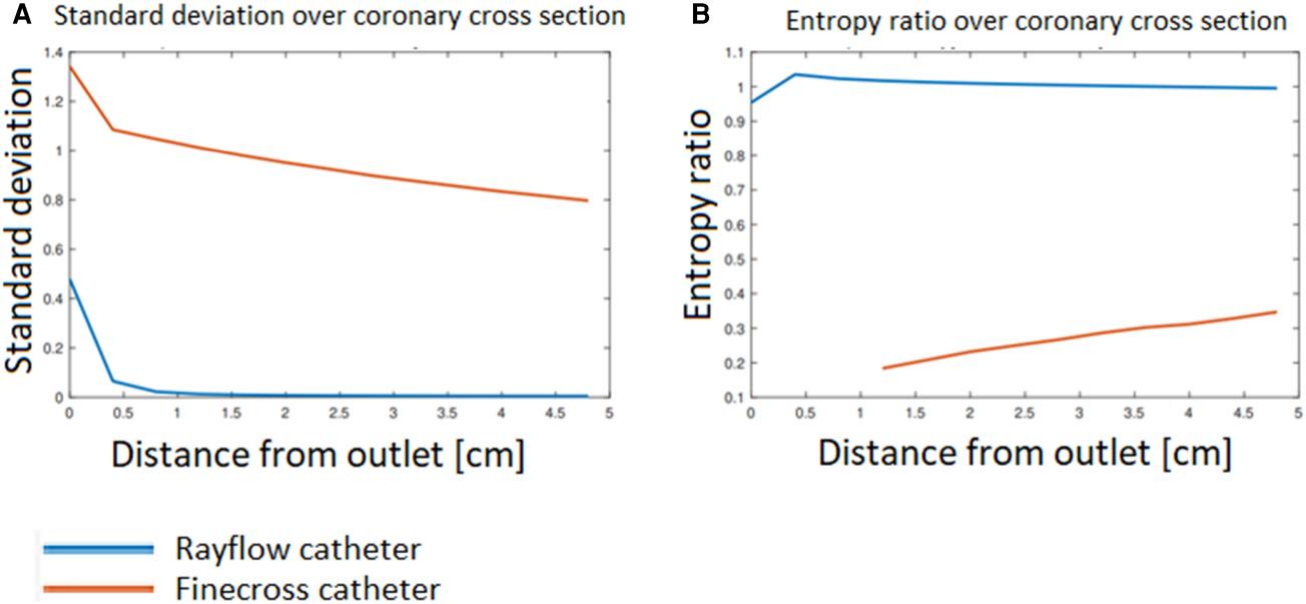
(*A*) Time-averaged standard deviation (SD_t_) and entropy ratio (*E*) (*B*) at different distances from the outlet during infusion for a simulation of coronary blood flow of 150 mL/min and an infusion rate of 15 mL/min for both the Rayflow (being the bottom line in panel A and upper line in panel B) and Finecross (being the upper line in panel A and the lower line in panel B) infusion catheters.

To visualize the course of the measures, SD_t_ and *E* distal to the infusion site values were calculated between the outlet and 4.8 cm into the coronary artery for a simulation of a MEDIUM coronary blood flow (150 mL/min) and an infusion rate of 15 mL/min. The results are shown in *Figure 6A* and *B* for the SD_t_ and *E*, respectively. The SD_t_ rapidly approaches 0 at around 1 cm distal to the infusion site for the Rayflow catheter. The course of the entropy ratio shows that the ratio approaches 1 at 0.5 cm distal to the infusion sites of Rayflow catheter (*Figure 6A*). The value for *E* reaches a maximum of 0.36 for the Finecross catheter over the measured distance (*Figure 6B*). Visualizations of the temperature field from the CFD simulations for both catheters can be found in the supplementary material.

## Discussion

In this study, it was shown that the mixing properties of the Rayflow catheter are superior compared with those of the Finecross catheter, a standard end-hole infusion catheter. The results were consistent throughout the qualitative, semi-quantitative, and quantitative methods. Especially in the clinically relevant combinations of infusion rate and coronary blood flow values, the Rayflow catheter is superior with respect to the mixing of blood and the injected fluid.

These experiments comprise the first systematic effort to assess the mixing properties of the dedicated Rayflow infusion catheter and compare them with a standard end-hole infusion catheter. In a previous *in vitro* study, it was concluded that the level of mixing was adequate since the application of continuous infusion thermodilution resulted in satisfactory agreement of coronary flow measurements compared with a gold standard for quantitative flow measurement.^4^ Similarly, *in vivo* experiments were performed across different animal models showing good agreement of the continuous infusion thermodilution and the gold standard perivascular flow probes.^9^ Clinical measurements have focused on the repeatability of the thermodilution technique to assume that the mixing was adequate as well in the absence of a true golden standard of flow measurement.^1^ None of these previous studies focused specifically on visualization of the mixing or determined (semi-)quantitative measures to assess mixing apart from one qualitative effort using an ink injection.^4^

In this study, all combinations of coronary flow rates and infusion rates have been examined systematically with a focus on the clinically relevant combinations. The three different methods give strong evidence that the specific design of the Rayflow catheter facilitates mixing in contrast to an infusion catheter with a single end hole. Despite the fact that applying a binary qualification (‘pass’ or ‘fail’) for the mixing patterns could yield ambiguity for the classification result, the superiority of the Rayflow is clear with respect to the adequacy of mixing. Both (semi-)quantitative methods support this conclusion, showing that mixing is excellent over the diameter of the coronary artery reflected by both the standard deviation values (SD_t_) near 0 and the entropy ratio (*E*) values near 1 in all the coronary flow and infusion rate combinations for the Rayflow catheter. The mixing was homogeneous over the length of the coronary vessel as well, quantified by the small number of data points that deviated more than 15–20% from the mean temperature along the pullback. On the contrary, for the Finecross catheter, a relative higher temperature was observed distal from the injection site and a lower temperature near the injection site indicating less homogeneous mixing. Based on the CFD analyses, one could conclude that if the sensor lies outside the ‘cold core’, the temperature will be close to the blood temperature resulting in a relative high distal temperature. Conversely, the temperature close to the tip of the infusion catheter is low which could be the result of the fact that the sensor is pulled through the ‘cold core’. The results of the non-clinically relevant combinations also support this conclusion as shown in Appendix 1.

As a limitation of this study, one could argue that the models of the coronary artery and infusion catheters are not exemplary for the coronary artery. All experiments were designed to approach the human coronary artery as closely as possible, but any model is a simplification of reality. For the CFD analyses, the characteristics of the blood were chosen to be equal to the infused fluid thereby neglecting the difference of viscosity and thermal properties. We purposely chose to simplify the 3D CFD model to mitigate the increased computational complexity and cost that this would entail. Moreover, the *in vitro* experiments complemented the CFD results in this way where the increased viscosity of the blood was taken into account.

Vice versa, the *in vitro* experiments are complemented by the CFD results for the qualitative assessment of the still frames. A ‘pass’ was defined when a uniform colour over the diameter was observed within 1–3 cm from the tip, but a proper assessment over the entire diameter is difficult since the vessel is filmed from only one side. The still frames miss information of the ink distribution over the vessel cross-section. Both the SD_t_ and the *E* from the CFD analyses represent the differences of the uniformity of the mixture over the cross-section and thus supplement the results from the qualitative assessment. Although the visual assessment for proper mixing could be a source of subjectivity, the differences between the catheter types remain obvious.

A physiological geometry of the coronary vessel, the compression of the coronary artery due to the spasm or contraction of the heart, and changes in vascular tone were not taken into account in the *in vitro* experiments and 3D CFD simulations. Similarly, the effect of increased or decreased viscosity (due to e.g. anaemia) was not studied either. However, the different models and measurements mimicked the general characteristics of the coronary artery and physiology as closely as possible and complemented each other. The effect of the location of the infusion catheter was indirectly studied because the experiments were repeated for five catheters of each type for the ink injections and for two catheters of each type for the temperature measurements. Stratifying the results by each catheter of both the Rayflow and Finecross infusion catheter yielded the same results. The compression of the artery could affect the blood flow in the coronary artery because of vortices and secondary flow patterns, which subsequently influence the mixing.^10^ These more realistic fluid dynamics would be beneficial for the mixing, but we believe that the superiority in mixing capabilities of the Rayflow catheter as shown in the results of this study will not diminish by these effects.

## Conclusions

In this study, we demonstrated that the specific design of the Rayflow catheter leads to homogeneous mixture of blood and saline over both the diameter and the length of a coronary vessel. These mixing properties are already nearly optimal near the tip of the catheter and superior over the entire range of coronary flow values and infusion rate values.

## Supplementary Material

ztae033_Supplementary_Data

## Figures and Tables

**Figure A1:**
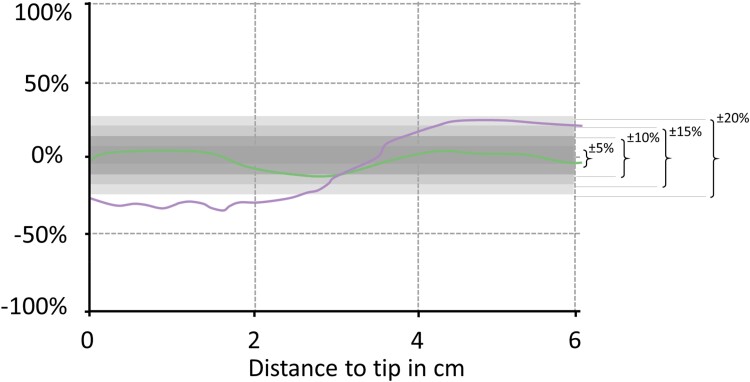
Example of percentage of data points in predefined ranges.

**Figure A2:**
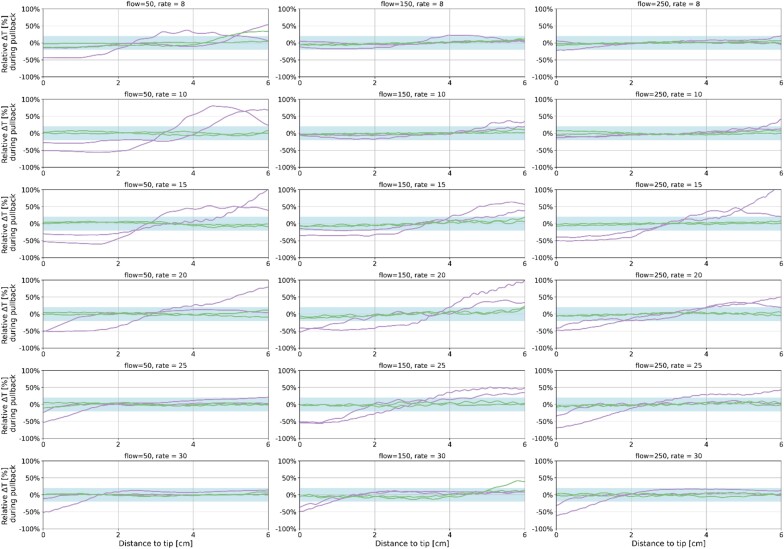
Relative deviations of the normalized measurements of temperature pullbacks for each combination of coronary flow rate and infusion rate for both catheters (Rayflow catheter in green and Finecross catheter in purple). The blue bar represents an area of 20% deviation, which is considered clinically acceptable.

## Data Availability

The data underlying this article will be shared on reasonable request to the corresponding author.

## Appendix

### Appendix 1

This appendix contains extra information, tables, and figures from the main article in order to clarify and explain our findings.

Results of *in vitro* setup:

Explanation and example of semi-quantitative assessment of mixing by temperature measurements

In the analyses to assess the homogeneity of temperature over the length of the vessel after mixing, 200 sample points of the temperature signal were obtained during pullback of the sensor. These sample points were normalized and expressed as % deviation from the mean temperature. This means that direct and homogenous mixing of the fluids would lead to stable—i.e. homogenous—temperature samples over the length of the pullback. The relative deviation of data points would then be close to 0% (example in *Figure 5* in the main article). Next, for each catheter, the percentage of data points that fall outside predefined ranges was calculated and shown in *Table 2*. *Figure 1* shows an example of data points and the predefined ranges.

**Table A1:** Results of visual fail/pass analysis of both Finecross® catheter (FC) and Rayflow® catheter (RF) at respectively 5 and 10 s after start of infusion, combined results for all combinations of coronary blood flow, and infusion rates

	5 s			10 s		
Catheter	Fail	Pass	Total	Fail	Pass	Total
FC	78	12	90	78	12	90
RF	30	60	90	23	67	90

*P*-value < 0.0001 in favour of Rayflow catheter for both time-frames.

### Appendix 2

Mesh sizes

Prescribed mesh settings:

- Mesh type: polyhedral
- Surface mesh:
  - Edge length:
    - Minimum: 5e^−5^
    - Maximum: 2.5e^−4^
    - Growth rate: 1.2
- Inflation layer:
  - Placed at:
  - Coronary wall
  - PT wire wall
  - Catheter walls (both sides)
  - Number of layers: 3
  - Transition ratio: 0.272
  - Growth rate: 1.2
- Volume mesh:
  - Edge length:
    - Maximum: 5e^−4^
    - Growth rate: 1.2

**Table A2:** Percentage of data points that fall outside the predefined ranges of acceptable relative deviation of temperature measurements, all combinations of blood flow, and infusion rates combined

	5%	10%	15%	20%
**Finecross**	65.1	50.4	39.9	31.1
**Rayflow**	21.9	4.0	1.1	0.7

FC, Finecross catheter; RF, Rayflow catheter.
