# Supplementary material for: Mixing properties of coronary infusion catheters assessed by in vitro experiments and computational fluid dynamics
Source: Eur Heart J Digit Health. 2024 May 16;5(4):491–501. doi: 10.1093/ehjdh/ztae033 (PMC11284010; doi:10.1093/ehjdh/ztae033)
Supplement: ztae033_Supplementary_Data [file ztae033_supplementary_data.zip › Supplement.docx]

**Supplemental material: legend to supplementary video’s**

Visualization of the Computational Fluid Dynamics (CFD) simulations. The temperature field is shown for a cross-section over the length of the mesh for the Rayflow (Rayflow_CFD.mp4) and the Finecross (Finecross_CFD.mp4) catheter. The CFD geometries are shown in Figure 2 of the main article with the pressure/temperature wire inside the Rayflow catheter and alongside the Finecross catheter.

Two heart cycles are simulated for each catheter with a coronary flow of 150 ml/min and an infusion rate of 15 ml/min.
